# A cross-species alignment tool (*CAT*)

**DOI:** 10.1186/1471-2105-8-349

**Published:** 2007-09-19

**Authors:** Heng Li, Liang Guan, Tao Liu, Yiran Guo, Wei-Mou Zheng, Gane Ka-Shu Wong, Jun Wang

**Affiliations:** 1 Beijing Institute of Genomics of Chinese Academy of Sciences, Beijing Genomics Institute, Beijing 101300, China; 2 James D. Watson Institute of Genome Sciences of Zhejiang University, Hangzhou 310008, China; 3 Institute of Theoretical Physics, Chinese Academy of Sciences, Beijing 100080, China; 4 Graduate University of the Chinese Academy of Sciences, Yuquan Road 19A, Beijing 100039, China; 5 Institute of Computing Technology, Chinese Academy of Science, Beijing 100080, China; 6 UW Genome Center, Department of Medicine, University of Washington, Seattle, WA 98195, USA; 7 The Institute of Human Genetics, University of Aarhus, DK-8000 Aarhus C, Denmark; 8 Department of Biochemistry and Molecular Biology, University of Southern Denmark, DK-5230, Odense M, Denmark

## Abstract

**Background:**

The main two sorts of automatic gene annotation frameworks are *ab initio *and alignment-based, the latter splitting into two sub-groups. The first group is used for intra-species alignments, among which are successful ones with high specificity and speed. The other group contains more sensitive methods which are usually applied in aligning inter-species sequences.

**Results:**

Here we present a new algorithm called *CAT *(for Cross-species Alignment Tool). It is designed to align mRNA sequences to mammalian-sized genomes. *CAT *is implemented using C scripts and is freely available on the web at .

**Conclusions:**

Examined from different angles, *CAT *outperforms other extant alignment tools. Tested against all available mouse-human and zebrafish-human orthologs, we demonstrate that *CAT *combines the specificity and speed of the best intra-species algorithms, like *BLAT *and *sim4*, with the sensitivity of the best inter-species tools, like *GeneWise*.

## Background

Gene annotation is often done by alignment of mRNAs to genome sequences. Compared to *ab initio *gene finding [1], this method is more reliable and avoids the need for training. The primary limitation is that not every gene will have an mRNA, but this problem will diminish as the databases grow, even if it never completely disappears. For intra-species alignments, there are many successful algorithms like *BLAT *[2] and *sim4 *[3]. They are known for their specificity and speed, even in mammalian-sized genomes, but they do not have the sensitivity that is needed for inter-species alignments. Other algorithms like *GeneWise *[4] do have the requisite sensitivity, but they are extremely slow. Hence, we developed a new algorithm called ***CAT ***(for Cross-species Alignment Tool) to combine the specificity and speed of the best intra-species algorithms with the sensitivity of the best inter-species algorithms.

From a technical perspective, there are three issues. First, sequence comparisons can be made at nucleotide or protein level. Examples of the former are *BLAT*, *sim4*, *est_genome *[5], and *exonerate *[6], while examples of the latter are *exonerate-aa *and *GeneWise*. Although it is believed that protein comparisons are intrinsically better at detecting distant homologies, we will show that this need not be the case. The real difference is that protein comparisons are better at determining exon-intron boundaries, since they can incorporate phase information. However, for aligning non-coding un-translated regions (UTRs), nucleotide comparisons are the only option. The second issue has to do with the tradeoffs between dynamic programming (DP) and heuristic methods. A full DP implementation like *est_genome *and *GeneWise *is good for specificity and sensitivity, but not for speed. Heuristic methods have been developed to increase the speed, often with a sacrifice in specificity and/or sensitivity, although as we will show, this too need not be the case. *BLAST *[7] is a well-known example, but *BLAT*, *sim4*, *exonerate*, and *exonerate-aa *also qualify. Such methods are most readily applied to nucleotide comparisons. The third issue is a consequence of the fact that one must allow for frequent gaps and mismatches to accommodate distant homologies in inter-species alignments; but in doing so, one increases the likelihood of false alignments. These typically appear as poorly matched terminal exons, a long distance from the end of the true alignment, and must be removed by statistical rules.

*CAT *is a nucleotide level alignment tool that uses improved heuristics to effectively balance specificity, sensitivity, and speed. It is designed for both intra-species and inter-species alignments. *CAT *can be freely downloaded at the website .

## Results

### Programs and test data set

We benchmarked *CAT *(version 0.8.2) against the following algorithms: *BLAT *(version 27), *sim4 *(version 2003-09-21), *GeneWise *(version 2.2.0), *est_genome *(located in *EMBOSS *[8] version 2.6.0), *exonerate *and *exonerate-aa *(version 0.8.2). Two comparisons were done. First, we aligned mouse mRNAs to human genome sequences, and then zebrafish mRNAs to human genome sequences. All the sequence data, including mRNAs, exon coordinates and genomes, were taken from the UCSC Genome Browser [9] (version hg16). Some of the mRNAs were flagged by UCSC because they mapped to more than one locus, and these were discarded. Ortholog relations came from *HomoloGene *[10] (version 2003-12-08). To ensure that we know what the "correct" answer is, we required all our human genes to have a mRNA in *RefSeq *[11] (version 2003-12-08). The final data set had 10,395 mouse-human and 2,007 zebrafish-human gene pairs.

### Definition of performance

The exact human mRNA coordinates, or alignments, on the human genome were given by UCSC. We arbitrarily took these alignments as the reference alignments, expecting most of them and their exon junctions could be recovered by aligning mouse (or zebrafish) orthologous mRNAs against the human genome. Although orthologous mRNAs in different species may differ at a few exons for a particular mRNA, a good overall agreement on the 10,395 orthologous pairs must indicate the good performance of a program. This is the basic assumption in our benchmark.

When we know what the correct answer is, performance can then be evaluated in the traditional manner [12]. We define true positive (TP), false positive (FP), and false negative (FN) at the nucleotide level as follows.

TP = number of aligned bases that overlap with the orthology annotation

FP = number of aligned bases that do not overlap with the orthology annotation

FN = number of bases in the orthology annotation that remain unaligned

At the nucleotide level, sensitivity (*nSn*) and specificity (*nSp*) are the proportion of correctly aligned bases with respect to the known and predicted alignment, respectively.

nSn=TPTP+FN
 MathType@MTEF@5@5@+=feaafiart1ev1aaatCvAUfKttLearuWrP9MDH5MBPbIqV92AaeXatLxBI9gBaebbnrfifHhDYfgasaacH8akY=wiFfYdH8Gipec8Eeeu0xXdbba9frFj0=OqFfea0dXdd9vqai=hGuQ8kuc9pgc9s8qqaq=dirpe0xb9q8qiLsFr0=vr0=vr0dc8meaabaqaciaacaGaaeqabaqabeGadaaakeaacqWGUbGBcqWGtbWucqWGUbGBcqGH9aqpdaWcaaqaaiabdsfaujabdcfaqbqaaiabdsfaujabdcfaqjabgUcaRiabdAeagjabd6eaobaaaaa@398B@

nSp=TPTP+FP
 MathType@MTEF@5@5@+=feaafiart1ev1aaatCvAUfKttLearuWrP9MDH5MBPbIqV92AaeXatLxBI9gBaebbnrfifHhDYfgasaacH8akY=wiFfYdH8Gipec8Eeeu0xXdbba9frFj0=OqFfea0dXdd9vqai=hGuQ8kuc9pgc9s8qqaq=dirpe0xb9q8qiLsFr0=vr0=vr0dc8meaabaqaciaacaGaaeqabaqabeGadaaakeaacqWGUbGBcqWGtbWucqWGWbaCcqGH9aqpdaWcaaqaaiabdsfaujabdcfaqbqaaiabdsfaujabdcfaqjabgUcaRiabdAeagjabdcfaqbaaaaa@3993@

At the exon level, sensitivity (*eSn*) and specificity (*eSp*) are defined as follows.

eSn=correctly aligned exonsexons in known alignment
 MathType@MTEF@5@5@+=feaafiart1ev1aaatCvAUfKttLearuWrP9MDH5MBPbIqV92AaeXatLxBI9gBaebbnrfifHhDYfgasaacH8akY=wiFfYdH8Gipec8Eeeu0xXdbba9frFj0=OqFfea0dXdd9vqai=hGuQ8kuc9pgc9s8qqaq=dirpe0xb9q8qiLsFr0=vr0=vr0dc8meaabaqaciaacaGaaeqabaqabeGadaaakeaacqWGLbqzcqWGtbWucqWGUbGBcqGH9aqpdaWcaaqaaiabdogaJjabd+gaVjabdkhaYjabdkhaYjabdwgaLjabdogaJjabdsha0jabdYgaSjabdMha5jabbccaGiabdggaHjabdYgaSjabdMgaPjabdEgaNjabd6gaUjabdwgaLjabdsgaKjabbccaGiabdwgaLjabdIha4jabd+gaVjabd6gaUjabdohaZbqaaiabdwgaLjabdIha4jabd+gaVjabd6gaUjabdohaZjabbccaGiabdMgaPjabd6gaUjabbccaGiabdUgaRjabd6gaUjabd+gaVjabdEha3jabd6gaUjabbccaGiabdggaHjabdYgaSjabdMgaPjabdEgaNjabd6gaUjabd2gaTjabdwgaLjabd6gaUjabdsha0baaaaa@6F96@

eSp=correctly aligned exonsexons in predicted alignment,
 MathType@MTEF@5@5@+=feaafiart1ev1aaatCvAUfKttLearuWrP9MDH5MBPbIqV92AaeXatLxBI9gBaebbnrfifHhDYfgasaacH8akY=wiFfYdH8Gipec8Eeeu0xXdbba9frFj0=OqFfea0dXdd9vqai=hGuQ8kuc9pgc9s8qqaq=dirpe0xb9q8qiLsFr0=vr0=vr0dc8meaabaqaciaacaGaaeqabaqabeGadaaakeaacqWGLbqzcqWGtbWucqWGWbaCcqGH9aqpdaWcaaqaaiabdogaJjabd+gaVjabdkhaYjabdkhaYjabdwgaLjabdogaJjabdsha0jabdYgaSjabdMha5jabbccaGiabdggaHjabdYgaSjabdMgaPjabdEgaNjabd6gaUjabdwgaLjabdsgaKjabbccaGiabdwgaLjabdIha4jabd+gaVjabd6gaUjabdohaZbqaaiabdwgaLjabdIha4jabd+gaVjabd6gaUjabdohaZjabbccaGiabdMgaPjabd6gaUjabbccaGiabdchaWjabdkhaYjabdwgaLjabdsgaKjabdMgaPjabdogaJjabdsha0jabdwgaLjabdsgaKjabbccaGiabdggaHjabdYgaSjabdMgaPjabdEgaNjabd6gaUjabd2gaTjabdwgaLjabd6gaUjabdsha0baacqGGSaalaaa@75AC@

where an exon is said to be correctly aligned, if and only if the known alignment (intra-species alignment) and predicted one (inter-species alignment) match end-to-end, which means the exon boundaries are identical between them.

Depending on the circumstances, we compute sensitivity and specificity in two ways, counting only the coding region (CDS) or counting the entire transcript (CDS+UTR).

### Comparison of algorithms

**Figure 1:**
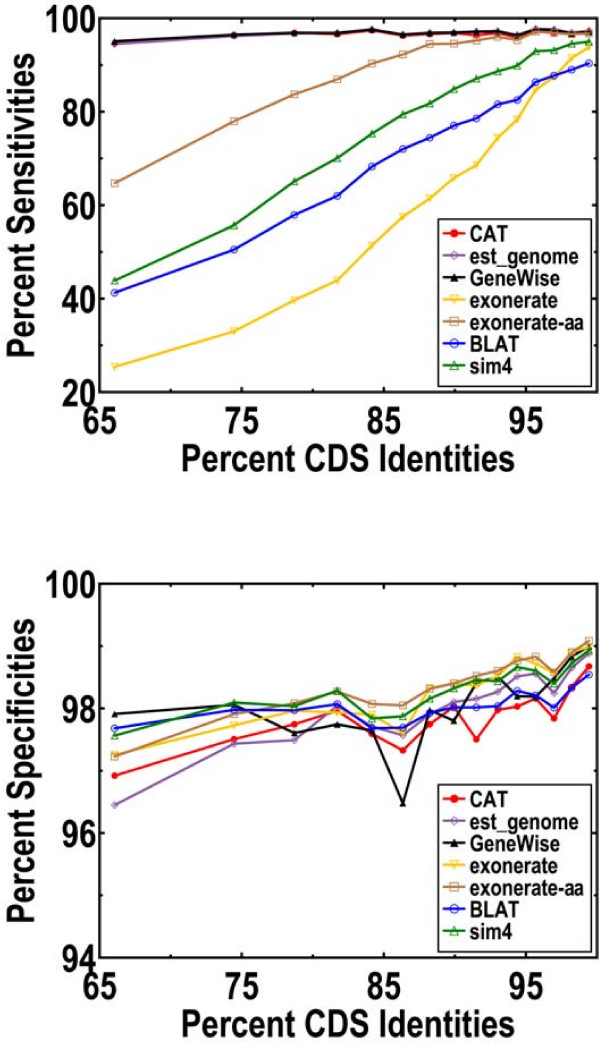
Nucleotide level sensitivity (*nSn*) and specificity (*nSp*). We restrict to coding regions, and display performance as a function of protein level identities in the aligned regions. Every data point represents 658 of the 10395 mRNAs from the mouse-human alignments. Obviously, the results for *CAT*, *est_genome *and *GeneWise *are hard to distinguish from each other when it comes to sensitivity. In plotting the figure, we discard the worst 5% of pairs where the fraction of aligned regions in respect to the length of full CDS is too small. These 5% of orthologous pairs tend to be wrongly predicted in the HomoloGene database due to their short aligned regions. Discarding them yields more consistent curves.

**Figure 2:**
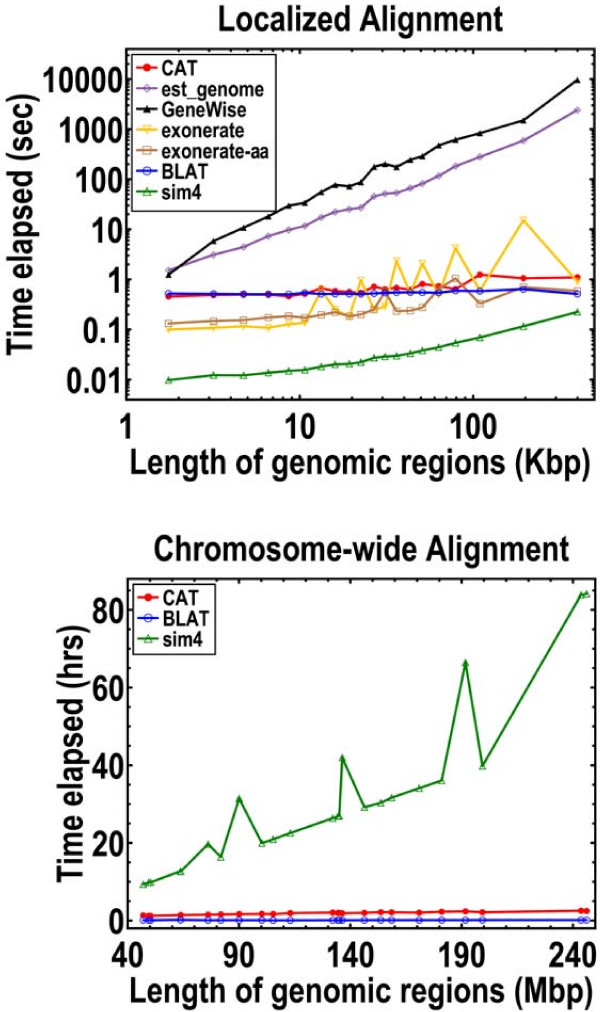
Speed comparisons for localized and chromosome-wide alignments. 1000 randomly selected mouse mRNAs are aligned against the human genome. In the localized plot, every data point represents the average of 50 alignments. In chromosome-wide plot, every data point is a single chromosome. This plot is limited to *CAT*, *BLAT*, and *sim4 *because they are the only ones that run in a reasonable amount of time and/or memory.

In our first set of measurements, we assume that each alignment can be localized to the orthologous region for that mRNA. All algorithms show comparably good specificities regardless of sequence divergence levels (Figure 1 and Table 1). *CAT*, *est_genome*, and *GeneWise *are the only ones that also show good sensitivities at the lowest CDS identities. If we insist that UTRs be included, then *CAT *and *est_genome *are by far the best algorithms. Although *sim4 *is the fastest (Figure 2 and Table 1), *CAT *is part of a group of moderately fast algorithms that includes *BLAT*, *exonerate*, and *exonerate-aa*. In contrast, *est_genome *and *GeneWise *are extremely slow, since they are the only ones to implement a full DP algorithm.

**Table 1:** Evaluation of localized alignments. 10395 mouse mRNAs and 2007 zebrafish mRNAs are aligned to the orthologous regions in the human genome.

**Mouse mRNA to Human genome**							
	CDS+UTR (nucl. level)		CDS alone (nucl. level)		CDS alone (exon level)		Speed (mRNA/hr)
Algorithm	Sn	Sp	Sn	Sp	Sn	Sp	
*CAT*	0.765	0.961	0.924	0.968	0.855	0.893	3579
*est2genome*	0.772	0.963	0.926	0.970	0.856	0.895	17
*GeneWise*	n/a	n/a	0.927	0.972	0.869	0.917	8
*Exonerate*	0.385	0.983	0.589	0.977	0.495	0.791	1254
*exonerate-aa*	n/a	n/a	0.856	0.977	0.787	0.890	10027
*BLAT*	0.487	0.976	0.678	0.973	0.161	0.172	5138
*BLAT-dnax*	0.615	0.979	0.872	0.975	0.513	0.518	1172
*sim4*	0.535	0.977	0.743	0.976	0.524	0.569	36815
							
**Zebrafish mRNA to Human genome**							
							
	CDS+UTR (nucl. level)		CDS alone (nucl. level)		CDS alone (exon level)		Speed (mRNA/hr)
Algorithm	Sn	Sp	Sn	Sp	Sn	Sp	

*CAT*	0.489	0.963	0.803	0.957	0.645	0.754	2806
*est2genome*	0.463	0.968	0.764	0.961	0.590	0.750	41
*GeneWise*	n/a	n/a	0.862	0.975	0.781	0.879	12
*exonerate-aa*	n/a	n/a	0.652	0.975	0.543	0.772	6757

The ''correct'' answers, against which we judge these algorithms, are based on an alignment of human mRNAs from *RefSeq *to the sequence of the human genome, as annotated in the UCSC browser.

However, it is not sufficient to require that the orthologous regions can be identified in a negligible amount of time. For annotation purposes, mRNAs must be aligned to mammalian-sized chromosomes in a reasonable amount of time. Of the studied algorithms, *CAT*, *BLAT*, and *sim4 *are the only ones where this condition is satisfied, but of these, only *CAT *can handle inter-species comparisons. *CAT *and *BLAT *are truly exceptional because their execution times scale nearly linearly with the size of the targeted genome sequences (Figure 2). All of the other algorithms scale very poorly with increasing size.

## Discussion

*CAT *is accelerated mainly in two ways. Firstly, *CAT *loads about 1000 mRNAs in one batch and scans genome once. Although the operations performed in scanning the genome are simple, doing these operations on a 3 Gb genome for 1000 times still amounts to a lot of computing time. *CAT *avoids unnecessary scans of genomes. Secondly, *CAT *chains high-scoring segment pairs (HSPs) localized in a window instead of on a whole chromosome. This allows *CAT *to find multiple hits on a chromosome and reduces the time spent on chaining. For *sim4*, chaining all the HSPs on a whole chromosome is the bottleneck of its speed.

*CAT *improves the sensitivity by using the techniques implemented in several previous softwares. Non-contiguous seeds [13], appropriate scoring matrix [14] and 2-round seeding [3] all help to achieve this goal. It is worth noting that localizing an mRNA to the top five windows may cause some true alignments to be missing. Fortunately, this happens rarely according to our practical observation in human-mouse alignments. For diverged species, this problem can also be largely avoided by retaining top 10 or more windows.

*CAT *uses a simple statistical test to improve the specificity in genome-wide alignments. As a matter of fact, most of false alignments are extremely short and poorly aligned. They typically appear at the first or last few exons. These false fragments are mainly due to random matches in seed finding, in cases where the true match is too divergent to be detected at our default thresholds. Only keeping statistically significant terminal exons, *CAT *is able to rule out most of false alignments.

As a rule, speed and sensitivity are in conflict with each other. Alignment algorithms must make a suitable compromise. Our data show that *CAT *sensitivity is competitive with algorithms like *est_genome *and *GeneWise*, which are based on a full dynamic programming implementation. The advantage is that *CAT *is much faster. It is not as fast as *BLAT *and *sim4*, but neither of these algorithms can compete with *CAT *on sensitivity. As to the utility of *CAT *for gene annotation, the reality of large-scale production projects is that mRNA sequencing cannot keep up with genome sequencing. In vertebrates, genome sequences have been released for human, chimpanzee, rhesus, mouse, rat, dog, chicken, tetraodon, and fugu. Many more are 'in the pipeline'. But, only in human [15], and especially in mouse [16], is there anything approaching a comprehensive collection of mRNA sequences. This discrepancy can be attributed to the intrinsic difficulty of extracting fresh mRNAs from the full complement of tissues, under all possible developmental and physiological conditions. More generally, for many genes in many species, the only mRNAs will probably be from another species. *CAT *therefore fulfills a need for practical gene annotation.

## Conclusion

Existing intra-species alignment algorithms, like *BLAT *and *sim4*, have relatively low sensitivity, while existing inter-species alignment tools, like *GeneWise*, fail to process sequences in a high-throughput style.

*CAT *offers an improved process that aligns mRNA sequences to mammalian-sized genomes. With respect to the performance of alignment, it achieves a winning combination of the specificity and speed of the best intra-species algorithms, like *BLAT *and *sim4*, with the sensitivity of the best inter-species tools, like *GeneWise*. Given how large-scale production of mRNA sequences tends to lag behind large-scale production of genome sequences, and how for certain genes the only available mRNAs are from another species, *CAT *fulfills a growing need for reliable genome annotation rooted in the experimental evidence of a real transcript.

## Methods

### Overview of *CAT *algorithm

**Figure 3:**
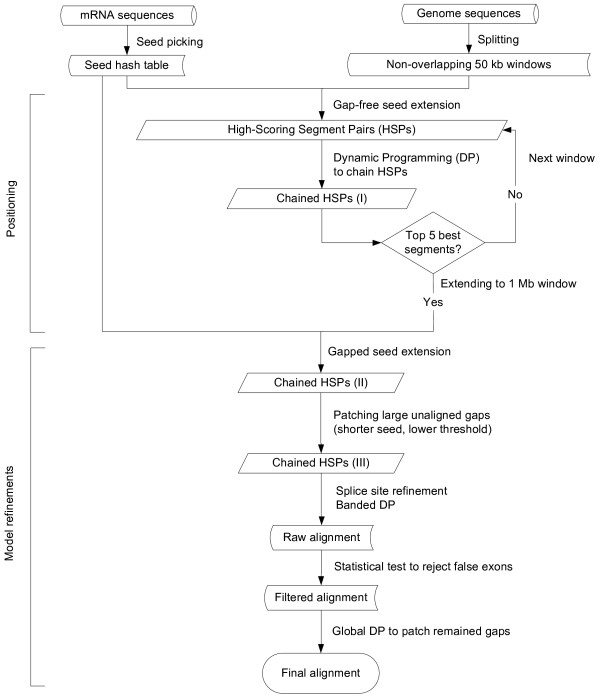
Flowchart of *CAT *algorithm (description in text of manuscript).

*CAT *is adapted from *sim4*, *BLASTZ *[17], and *PatternHunter *[13]. It uses the seed-extension strategy first introduced in *BLAST*, but with some important differences, like multiple rounds of seeding and seeds that need not be contiguous. Together with the scoring matrix by Chiaromonte *et al*. [14], these algorithmic changes provide the requisite improvement in sensitivity to perform cross-species alignments. Speed is improved by first localizing the mRNA to small windows and then restricting the time consuming procedures to these windows. This allows *CAT *to run on mammalian-sized genomes. Here, we describe the basic idea (Figure 3).

1. Read the whole genome into memory.

2. Get, typically, 1000 mRNAs and build a seed hash table.

3. Divide genome into non-overlapping 50 Kb windows. Scan windows for seeds in the hash table, and then perform a gap free seed extension at each hit. Calculate aligned lengths for all the HSPs.

4. Join HSPs by dynamic programming (DP) method, keeping the five windows with the highest total aligned lengths, and discarding all the others.

5. Expand windows to 1 Mb. Patch small gaps in a new round of seeding and extension, discarding conflicting alignments as required.

6. Patch large gaps using smaller seeds and lower thresholds.

7. Identify splice sites with banded DP algorithm.

8. Remove false exons using statistical test described below.

9. Do global alignment to close remaining gaps.

Detailed description of each step follows:

### Loading data

*CAT *keeps the genome sequences in the physical memory and loads, typically, about 1000 mRNAs each time in the main loop. It then scans all the mRNAs nucleotide by nucleotide, extracts the sequence according the seeding template [17], and stores the coordinate in a hash table based on the extracted sequence. In *CAT*, the default template is 111010010100110111 [13].

### Localizing mRNA

*CAT *then scans the whole genome window by window and searches for seeding hits against the hash table. The initial hits are extended without gaps by maximizing the alignment score. Only high-scoring ones (HSPs [2]) are retained. *CAT *performs a dynamical programming to find, in each window, the collinear HSP chain which gives longest alignment length. For each mRNA, the length of the chain and the coordinate of the window are maintained in a heap data structure where only the best 5 windows are retained.

### Constructing backbone alignments

For each mRNA and each 50 Kb window stored in the heap, *CAT *extends the window to 1 Mb, putting the 50 Kb window in the center. Adjacent 50 Kb windows are merged to avoid overlaps in window extension.

In a new 1 Mb window, seeding, gap-free extension and HSP chaining is performed again because HSP coordinates are not stored in previous steps in attempt to reduce the memory usage. Furthermore, in the mRNA regions where no hit is found, a second round of seeding-extending-chaining is applied to patch gaps. The seeding template used in this round is 11011011, which actually captures the fact that the first two nucleotides in a codon tend to be more conservative. This 2-round alignment can increase sensitivity and has been used in both *sim4 *and *BLASTZ*.

### Constructing raw exon alignments

The backbone alignment is actually a chain of gap-free fragments. A banded affine-gap Smith-Waterman [18] is applied to extend each fragment at both 5'- and 3'- ends. If two adjacent fragments are overlapped with each other after the Smith-Waterman extension, they will be joined together to form a longer gapped segments.

Like *sim4*, *CAT *determines exon boundaries in a heuristic way. If two adjacent extended segments have overlaps, *CAT *tries to find the break points where there is a GT-AG signal; if the two segments have no overlap, *CAT *looks for GT-AG 7 bp ahead and adds gaps arbitrarily to meet the splicing signal. If no GT-AG signals can be found anyway, *CAT *will arbitrarily choose a splicing sites without the signal.

### Refining alignments

For exons with gaps added at the ends to meet GT-AG signals, a banded affine-gap Needdleman-Wunsch algorithm is further applied to pinpoint the positions of these gaps. After that, *CAT *rules out low-confidence terminal exons based on the statistical model described below. If there are still unaligned regions between adjacent exons, *CAT *will perform an adapted global alignment to close the gaps on mRNAs. This adapted algorithm adds an "intron state" to the original global alignment. Its recursion functions resemble the ones used in *EXALIN *[19]. The resultant alignment will be output as the final results.

### Statistics of terminal exons

According to the central limit theorem, we can approximate the probability *q*(*l*, *s*) for a gap-free alignment of length *l *and score *s*:

q(l,s)=Pr⁡{S>s}=12erfc(s−μlσl)
 MathType@MTEF@5@5@+=feaafiart1ev1aaatCvAUfKttLearuWrP9MDH5MBPbIqV92AaeXatLxBI9gBaebbnrfifHhDYfgasaacH8akY=wiFfYdH8Gipec8Eeeu0xXdbba9frFj0=OqFfea0dXdd9vqai=hGuQ8kuc9pgc9s8qqaq=dirpe0xb9q8qiLsFr0=vr0=vr0dc8meaabaqaciaacaGaaeqabaqabeGadaaakeaacqWGXbqCcqGGOaakcqWGSbaBcqGGSaalcqWGZbWCcqGGPaqkcqGH9aqpcyGGqbaucqGGYbGCcqGG7bWEcqWGtbWucqGH+aGpcqWGZbWCcqGG9bqFcqGH9aqpdaWcaaqaaiabigdaXaqaaiabikdaYaaacqWGLbqzcqWGYbGCcqWGMbGzcqWGJbWycqGGOaakdaWcaaqaaiabdohaZjabgkHiTGGaciab=X7aTnaaBaaaleaacqWGSbaBaeqaaaGcbaGae83Wdm3aaSbaaSqaaiabdYgaSbqabaaaaOGaeiykaKcaaa@50DE@

In the above formula, *erfc*() is the complement of the error function, μ_*l *_is the mean, and σ_*l *_is the variance of the scores of random gap-free alignments of length *l*. Both μ_*l *_and σ_*l *_are calculated from the scoring matrix.

On the assumption that we know an exon alignment *e*_*1 *_is correct, we can calculate the probability *p*(*l, s, d*) for finding an adjacent exon alignment *e*_*2 *_(with length *l *and score *s*) in a distance at most *d *from *e*_*1*_:

*p*(*l*, *s*, *d*) = Pr{*D *≤ *d *} = 1-(1-*q*(*l*, *s*))^*d*^

We recognize that above formula discriminates against large introns, some of which might be real, but if we had not adopted such a formula, there would have been too many false alignments. Exons are allowed (kept in the final alignment) according to the following three rules.

1. Exons *e*_*1 *_satisfying *q*(*l*_*1*_*, s*_*1*_) <*t*_*1 *_are kept in the final alignment

2. When exon *e*_*1 *_is kept in the final alignment, exon *e*_*2 *_is kept if *p*(*l*_*2*_*, s*_*2*_*, d*) <*t*_*2*_

3. Exons between kept exons are also kept in the final alignment

**Figure 4:**
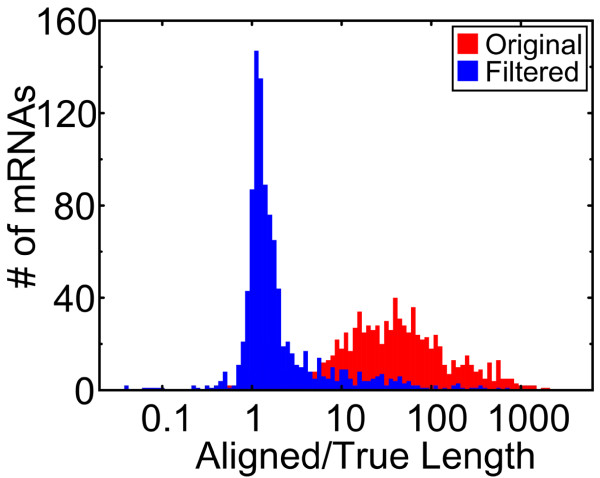
Statistical filtering of terminal exons. Here, 1000 randomly selected mouse mRNAs are aligned to the human genome. We show the ratio of aligned to true length, before (red) and after (blue) statistical filtering. Length refers to the extent of the mRNA alignment from the start codon to the stop codon. In other words, UTRs are excluded.

We use default settings of 10^-14 ^and 10^-9 ^for *t*_*1 *_and *t*_*2*_, respectively. The first rule ensures that if the alignment is significant on its own, that exon is naturally kept in the final alignment. The second rule says that, even if the alignment is not so significant on its own (*q(l*_*1*_*, s*_*1*_*) ≥ t*_*1*_), it will be kept in the final alignment if it is sufficiently close to another exon that has already been kept in the final alignment. The third rule ensures continuity. We remove terminal exons, not interior exons. A simple test demonstrates how effective this is (Figure 4).

## Availability and requirements

Project name: CAT (formerly XAT)

Project home page: 

Operating system: All POSIX (Linux/BSD/UNIX-like OSes)

Programming language: C

Other requrements: None

License: GNU General Public License (GPL)

Any restrictions to use by non-academics: None

## Competing interests

The author(s) declares that there are no competing interests.

## Authors' contributions

JW and GKSW brought the initial ideas and supervised the project. HL and WMZ developed the methods and algorithms. LG and HL implemented the software. TL collected data and evaluated the performance of different programs. HL and YG drafted the manuscript and WMZ and GKSW revised it. All authors have read and approved the final manuscript.
